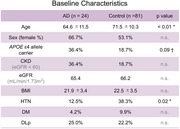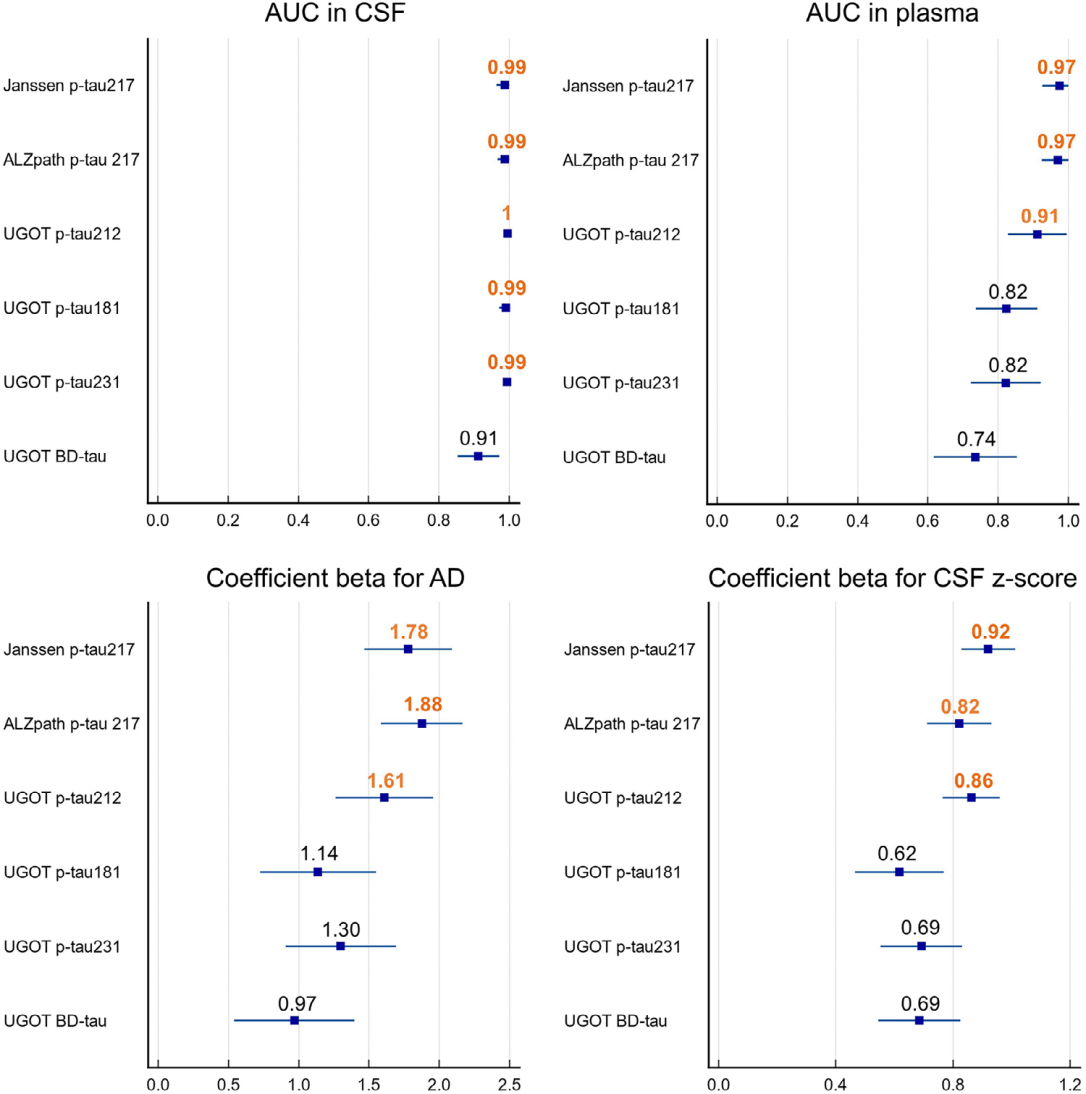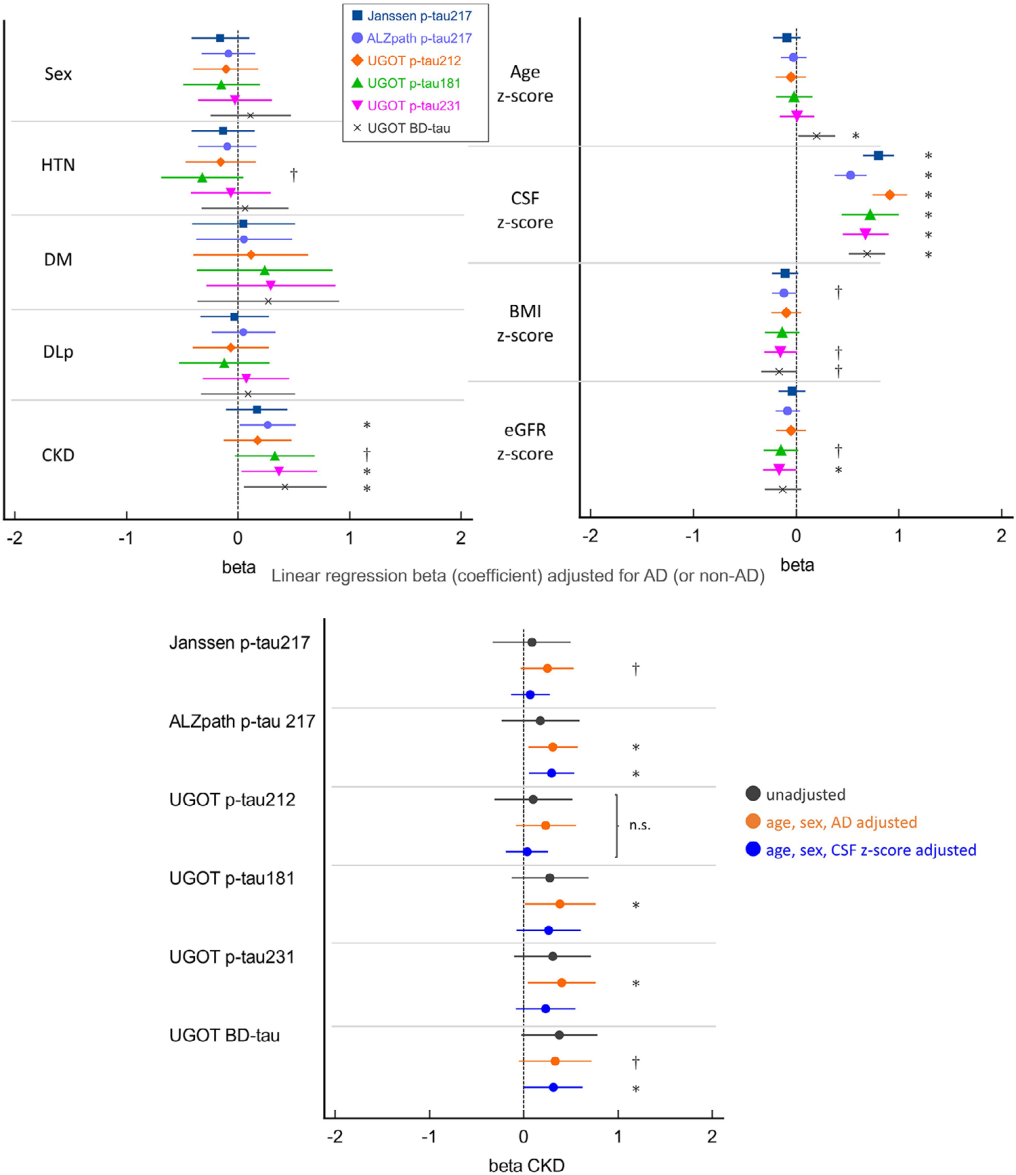# Comparison of renal effect on various plasma tau assays in reference to CSF values

**DOI:** 10.1002/alz70856_096833

**Published:** 2025-12-24

**Authors:** Masanori Kurihara, Nicholas J. Ashton, Przemyslaw Radoslaw Kac, Giovanni De Santis, Kenji Ishibashi, Kenji Ishii, Henrik Zetterberg, Atsushi Iwata, Kaj Blennow, Tokyo Medical Biobank Study Group

**Affiliations:** ^1^ Tokyo Metropolitan Institute for Geriatrics and Gerontology, Tokyo, Japan; ^2^ Department of Psychiatry and Neurochemistry, Institute of Neuroscience and Physiology, The Sahlgrenska Academy, University of Gothenburg, Mölndal, Gothenburg, Sweden; ^3^ Department of Psychiatry and Neurochemistry, Institute of Neuroscience & Physiology, the Sahlgrenska Academy at the University of Gothenburg, Mölndal, Gothenburg, Sweden; ^4^ Tokyo Metropolitan Institute for Geriatrics and Gerontology, Tokyo, Tokyo, Japan; ^5^ Department of Psychiatry and Neurochemistry, Institute of Neuroscience and Physiology, The Sahlgrenska Academy, University of Gothenburg, Mölndal, Sweden

## Abstract

**Background:**

Now that plasma tau assays show excellent diagnostic accuracy for AD, clinical implementation is important. Although renal function is known to affect values in some assays, it was unknown whether the effect size differs between assays in reference to CSF values.

**Method:**

Paired cerebrospinal fluid (CSF) and plasma samples from 105 donors of our biobank (24 symptomatic AD, 81 disease controls) were analyzed. Diagnoses of AD were confirmed by PET and/or validated CSF biomarkers. Disease controls included amyloid‐negative patients with mild cognitive impairment, frontotemporal dementia, and Lewy body disease. CSF and plasma Janssen *p*‐tau217, ALZpath *p*‐tau217, *p*‐tau181, *p*‐tau 231, *p*‐tau212, and BD‐tau were measured on the Simoa platform at the University of Gothenburg. Information on age, sex, renal function (eGFR), chronic kidney disease (CKD), and other comorbidities were obtained. Continuous variables were transformed to z‐score for comparison.

**Result:**

Baseline characteristics were similar between groups besides younger age, higher presence of *APOE* ε4 carrier, and lower presence of hypertension in AD (Table 1). All *p*‐tau assays showed high areas under the curve (AUCs) in the CSF and CSF BD‐tau showed AUC of 0.91. However, AUC was significantly different in the plasma; the highest AUCs were observed for *p*‐tau217 assays (AUC=0.97 [0.93–1]) followed by *p*‐tau212 (AUC=0.91 [0.83–1]). Linear regression analysis showed that beta for AD was highest for ALZpath *p*‐tau217 followed by Janssen *p*‐tau217 and *p*‐tau212. Beta for CSF z‐score was highest for Janssen *p*‐tau217 followed by *p*‐tau 212 suggesting better association with CSF (Figure 1). Regression analysis adjusted for AD or non‐AD showed higher beta for CKD in ALZpath *p*‐tau217, *p*‐tau181, *p*‐tau231, and BD‐tau. The association between plasma *p*‐tau212 and renal dysfunction remained non‐significant in several analyses (Figure 2). The association of plasma markers with age, sex, medical history of hypertension, diabetes mellitus, or dyslipidemia were not significant.

**Conclusion:**

We confirmed the trend towards higher values associated with CKD in most plasma tau assays. While *p*‐tau217 assays showed slightly higher AUCs and coefficient beta for AD, the high association with CSF values and possible lower association with renal dysfunction in *p*‐tau212 may warrant further investigation in larger studies.